# The Medical Uses of the *X*-Rays and Their Employment for These Purposes by Unqualified and Unskilled Persons

**Published:** 1904-05-14

**Authors:** 


### Hospital Clinics.

### THE MEDICAL USES OF THE X-RAYS AND THEIR EMPLOYMENT FOR THESE PURPOSES BY UNQUALIFIED AND UNSKILLED PERSONS.

It may not have occurred to many who have not followed the development of radio-therapeutic science that what is constantly being urged by experienced radio-therapeutists is anything more than a desire to retain in their own hands these methods of treatment. But a short inquiry into the subject will show clearly the advisability of the retaining of the therapeutic uses of the $x$-rays in hands of fully-qualified and experienced medical men. It is constantly coming before our notice that tradesmen and more scientific electricians are turning their instruments to account, whether at the instance of medical men, or because they have been desired to do so by the patients themselves in the treatment and diagnosis of disease. In no more important condition can the danger of this unskilled treatment be urged than in the management of a case of cancer. Unfortunately, so much has the subject been "written up" by the headlong and precipitate correspondent that the laity have an idea that the $x$-rays have taken the place of the alchemists' elixir vitæ, and all they have to do is to sit and be cured.

That cases of undoubted malignant disease have done well temporarily, is acknowledged on all hands, and instances have come under our own experience, but so also, unfortunately, has the reverse of this been obvious, where not only has no good been done, but where there has been considerable doubt as to whether the disease has not progressed more rapidly in consequence of the treatment. This being the case, as in advising a kill or cure operation, the circumstances of the case should be laid carefully before the patient, and the latter left to choose accordingly.

Again, there seems to be reason for supposing that the margin of treatment is a very narrow one. That is to say, with too little of the rays the whole effect may be stimulation of the growth, as well as of the healthy cells. With too much of the rays the healthy cells may be injured so that the disease may progress rapidly, and between these two there remains only a narrow mean wherein the safe administration can be expected to so depress the unhealthy cells that the healthy ones can overcome the diseased cells.

Considering how difficult this determination of the safe margin is, it is certainly of importance that no one but a skilled administrator should be entrusted with the case. The time is not yet ripe, if it ever will be, for the treatment of primary operable cases with the rays. Once the patient has fallen into the hands of the sciolist, the charlatan, or the quack, what chance will there be that he would recognise the indication for operation, or that he would recommend such a course if he did? The word "burn" is associated indissolubly with the $x$-ray treatment, and by it is meant a certain train of symptoms ensuing upon an over-dose of the rays. A case has recently come to our knowledge in which such a condition occurred in the practice of a medical man who had had little experience of the $x$-ray, and the "burn" was not recognised as such. How much more is there a possibility of such an occurrence in the hands of the local electrician and gasfitter? This may appeal to all, and yet it may be held that no harm can be done in screening cases for injury to and in the diagnosis of disease in bones. But Mr. Shenton has pointed out in the *Medical Electrological and Radiological Journal* how easy it would be for an unskilled observer to miss or estimate wrongly the appearance of an endosteal sarcoma: mistaking it for a fracture with ensuing callus formation. Undoubtedly experience counts for much in the working of an $x$-ray screen. An observer who is not frequently using the apparatus will miss entirely an important phenomenon and not be able to see such when pointed out to him, whereas a skilled observer would recognise easily conditions which cannot even be seen by others. This will later be seen to be of greater importance when the screen is used more, as it undoubtedly will be, in the diagnosis of diseases of the chest. Much more could be written upon this subject, but our intention is gained if we have stirred up some thought on what appears, even in medical circles, to be a callous and careless belief in the fetish of $x$-rays.

